# Maternal immune activation and role of placenta in the prenatal programming of neurodevelopmental disorders

**DOI:** 10.1042/NS20220064

**Published:** 2023-05-31

**Authors:** Rebecca M. Woods, Jarred M. Lorusso, Jennifer Fletcher, Heidi ElTaher, Francesca McEwan, Isabella Harris, Hager M. Kowash, Stephen W. D'Souza, Michael Harte, Reinmar Hager, Jocelyn D. Glazier

**Affiliations:** 1 Division of Evolution, Infection and Genomics, School of Biological Sciences, Faculty of Biology, Medicine and Health, Manchester Academic Health Science Centre, University of Manchester, Manchester M13 9PT, U.K.; 2 Division of Pharmacy and Optometry, School of Health Sciences, Faculty of Medicine, Biology and Health, Manchester Academic Health Science Centre, University of Manchester, Manchester M13 9PT, U.K.; 3 Department of Physiology, Faculty of Medicine, Alexandria University, Egypt; 4 Division of Developmental Biology and Medicine, School of Medical Sciences, Faculty of Biology, Medicine and Health, Manchester Academic Health Science Centre, University of Manchester, Manchester M13 9WL, U.K.

**Keywords:** cytokine, epigenetic regulation, fetal brain, infection, placenta, pregnancy

## Abstract

Maternal infection during pregnancy, leading to maternal immune activation (mIA) and cytokine release, increases the offspring risk of developing a variety of neurodevelopmental disorders (NDDs), including schizophrenia. Animal models have provided evidence to support these mechanistic links, with placental inflammatory responses and dysregulation of placental function implicated. This leads to changes in fetal brain cytokine balance and altered epigenetic regulation of key neurodevelopmental pathways. The prenatal timing of such mIA-evoked changes, and the accompanying fetal developmental responses to an altered *in utero* environment, will determine the scope of the impacts on neurodevelopmental processes. Such dysregulation can impart enduring neuropathological changes, which manifest subsequently in the postnatal period as altered neurodevelopmental behaviours in the offspring. Hence, elucidation of the functional changes that occur at the molecular level in the placenta is vital in improving our understanding of the mechanisms that underlie the pathogenesis of NDDs. This has notable relevance to the recent COVID-19 pandemic, where inflammatory responses in the placenta to SARS-CoV-2 infection during pregnancy and NDDs in early childhood have been reported. This review presents an integrated overview of these collective topics and describes the possible contribution of prenatal programming through placental effects as an underlying mechanism that links to NDD risk, underpinned by altered epigenetic regulation of neurodevelopmental pathways.

## Introduction

The aims of this review are to consider the molecular pathways by which infection in pregnancy, and prenatal exposure *in utero* to maternal immune activation (mIA), affects fetal neurodevelopment and might program neurodevelopmental disorders (NDDs), which manifest later in life. This involves an intricately complex set of signalling pathways that appear to be dysregulated following mIA and the ensuing maternal stimulation of cytokine and chemokine release. We present an integrated approach across the developmental timeline and focus on key mechanistic pathways that are likely to be involved and modulated by epigenetic changes. Importantly, we address mIA-associated changes in the maternal, placental and fetal compartments, with particular emphasis on neural development, along the longitudinal axis of signal transduction. Through this approach, we aim to delineate loci of dysregulated responses following maternal infection and evoked mIA, some of which may be maladaptive and developmentally enduring. We briefly consider the evidence for how maternal infection influences NDD risk, then discuss animal models of mIA and their validity, focussing on mIA models in mice and rats. We then examine the role of the placenta in the programming of NDDs through a cytokine-mediated pathway and associated inflammatory responses and explore what neurodevelopmental impacts this might have, and how the mIA-driven dysregulation of neurodevelopmental events is likely to involve epigenetic modulation. We finally discuss the translational significance of the collective scope of literature included in the review in the context of the recent COVID-19 pandemic and potential neurodevelopmental impairments and the possible underlying contribution of prenatal programming.

## Prenatal infection, maternal immune activation and neurodevelopmental disorders

During pregnancy, major physiological, endocrinological and immunological adaptations occur to ensure a successful outcome to pregnancy and optimal fetal development [1]. Understanding the molecular mechanisms of how these adaptations are orchestrated and regulated, particularly as regards immunological changes, is crucial if vaccines against microbial infections are to be developed to offer protection to both the pregnant mother and developing fetus [1,2]. Such knowledge is also vital if functional loci are to be identified for targeted interventions. These considerations are set against a background whereby an intricate immunological balance prevails that allows immunological tolerance to the semi-allogenic fetus but also protects both the mother and fetus against infectious pathogens [1,2]. Hence, a perturbed immunological environment during pregnancy, induced by a stressor such as maternal infection, can lead to increased risk of multiple pregnancy dysfunctions with potential impacts on fetal development [1,2].

Infectious pathogens are known to display seasonal variance, and it was noted that babies born in winter and spring months had an increased risk of developing schizophrenia (SZ) and other neuropsychiatric disorders when compared with the rest of the general population [3]. Indeed, many epidemiological studies investigating the prenatal exposure to the influenza epidemics of 1954, 1957 and 1959 reported a significant increase in adult SZ in the offspring of exposed mothers [4–6], with collective evidence supporting a well-established causal relationship between prenatal infection, particularly during the first trimester, and neurological disease of the offspring [7]. Another distinctive example supporting the association between prenatal infection and NDDs is the rubella epidemic of 1964. Prior to it, the incidence of NDDs such as autism spectrum disorders (ASD) and SZ was less than 1%, while subsequent to the epidemic, the incidence of ASD increased to 13% while that of SZ rose to 20% [8]. Additionally, outbreaks of measles, mumps, *Toxoplasmosis gondii*, as well as polio, cytomegalovirus and chickenpox have increased the incidence of NDDs [8–10]. However, the broad diversity of pathogens implicated in increasing NDD risk, involving both viruses and bacteria, suggest the causal link is not limited to a specific pathogen *per se*, but rather to a common immune response to these pathogens acting as a key driving force, with mIA proposed as the fundamental mediator between the prenatal infection and the resulting neuropathological changes in the offspring [8–13]. Indeed, many epidemiological studies have explored the detrimental effects of exposure to infection during pregnancy not only on the raised risk of maternal illness severity [14] but also the propensity to altered fetal neurodevelopment through mIA-driven pathways with the subsequent manifestation of NDDs, most notably ASD and SZ in the offspring [8,11,15–17].

While many animal and human studies support the hypothesis of ‘mIA as a mechanism for subsequent NDDs’ [9,13,18], it is worth commenting that many cases of prenatal infections, do not, however, result in the development of a NDD phenotype in the progeny. This suggests that mIA may act to ‘prime’ for NDDs, working with other synergizing risk factors or ‘hits’, such as genetic predisposition, abnormal immune status and simultaneous or subsequent exposure to other environmental challenges to induce neuropathological changes [8,11]. Hence, the core concept of the ‘multiple-hit paradigm of NDDs’ for disorders such as SZ [19,20], involves the interplay between infection, genetics and environment [21,22]. Within this paradigm, the propensity to develop NDDs arises from mIA-induced disruption of neurodevelopment and maturation of the brain in prenatal and early neonatal life, leading to programmed and lasting changes in behavioural development and an increased risk of developing SZ during late adolescence or early adulthood [23], with a similar concept modelled for ASD [24].

It is interesting to note that the risk that mIA imparts on neurodevelopmental outcomes [11] is not limited to neurodevelopmental psychiatric conditions such as SZ [10,25] and ASD [26–28] but also extends to attention-deficit hyperactivity disorder (ADHD) [29]. Moreover, while epidemiological research has drawn links between maternal infection, immune activity, and subsequent neurodevelopmental risk, these varied neurobehavioural outcomes illustrate that phenotypic outcomes are heterogeneous in nature, and likely to be influenced by a number of factors that dictate both the specificity and severity of disease. Such heterogeneity in neurodevelopmental sequelae may also reflect a lack of concordance in the developmental timing of infection [30,31] as well as a failure to consider concurrent environmental stressors that may inflate the relationship between the elicited immune response and offspring psychiatric outcome [32]. Another interpretation consistent with such outcomes is that a proportion of offspring exhibit an innate degree of resilience to mIA influences, with apparent protection from neurodevelopmental pathology, suggesting multiple factors interact to confer individual susceptibility to disease risk [30,33,34]. In behaviourally-susceptible offspring, for example, significant differences in gene expression in brain regions associated with NDDs are found, and subgroups can be additionally identified related to differences in offspring plasma cytokine concentrations of IL-6, TNFα, IL-1β and IL-10 [34], mirroring what can be seen in cases across clinical diagnostic criteria [35–37].

Notwithstanding this complexity, many etiopathological studies have focused on the causal mechanisms between gestational exposure to infection and the development of NDDs and neuropsychiatric disorders of the offspring, with an emergent recognition that maternal infection exposures exhibit some degree of synergism to other metabolic or environmental stressors during pregnancy, such as maternal nutritional deficiency or overnutrition, along with stress [38,39]. Hence, mIA-induced changes in fetal neurodevelopmental trajectory can be viewed broadly within the framework of the Developmental Origins of Health and Disease (DoHaD) paradigm, whereby stressors experienced *in utero* influence fetal developmental processes and trajectories through maladaptive changes that lead to aberrant health outcomes in the offspring [38].

## Preclinical models of mIA and neurodevelopmental disorders

The use of preclinical modelling of mIA, most extensively in mouse and rat models [15,18,40–42], has allowed wide-ranging investigation into the relationship between mIA and subsequent NDD risk, with greatest translational value bestowed when the central focus of investigation is clinically relevant behaviours and conserved molecular mechanisms [40]. Experimental modelling of mIA in mouse and rat models has good physiological and molecular translatability to human disorders, as these species share many neurochemical, neuroanatomical, transcriptomic and epigenetic changes in the offspring’s brain, which, along with abnormal behavioural phenotypes, recapitulate several traits seen in human NDDs [31,43,44]. Additionally, these models adequately recapitulate many features of human prenatal forebrain development at gestational landmarks of comparable relative temporality [40,44].

A further translational benefit of these mouse and rat mIA models is that in common to humans, all these species have haemochorial placentation (i.e. the placental trophoblast cells of the exchange barrier are in direct contact with maternal blood [45,46]), allowing some cross-species extrapolation when investigating placental changes arising from mIA-elicited immune responses, altered *in utero* environment, and the effects on placental function and ensuing influences on fetal brain development. They also afford the opportunity to examine the impacts induced by mIA on placental function, concomitant to the neural developmental and maturational processes occurring in the fetus, enabling the evolution of the pathogenesis of NDDs to be examined along a progressive ontogenetic timeline. Their further translational value is underpinned by the conservation of genes involved in placentation across these species [46–48], as well as an array of common molecular mechanisms of placental function and placental transport physiology [45,49–53], making them attractive models to mimic the programming of dysfunctional developmental processes of human pregnancy.

However, one distinction to note is that the placentas of mouse and rat are subdivided into two functional zones, the labyrinth zone (LZ) concerned with maternofetal nutrient exchange and the junctional (or basal) zone (JZ) which has an endocrine function [45–48]. Another important point worth raising, is in rodents such as mouse and rat, the visceral yolk sac (hereafter referred to as yolk sac) is retained throughout pregnancy [54,55], in contrast with the human yolk sac which undergoes regression towards 10-12 weeks of pregnancy [56], although both contribute to early fetal growth and development [54,56,57] and, further, transcriptomic analysis of yolk sac gene expression shows a high degree of conservation across these species [57]. We have recently identified the rat yolk sac as an immunoinflammatory and mIA-responsive tissue which expresses Toll-like receptor (TLR)3 and stimulates proinflammatory cytokine gene expression [43]. As the rodent yolk sac contributes to the maternofetal transport of nutrients, influencing fetal growth and developmental processes [43,54,55], and is the source of microglia progenitors that migrate and colonise the embryonic brain [58,59], this has relevance as the inflammatory stimulation of microglial activation is considered a risk factor for the development of NDDs, as discussed below. Interestingly, we have demonstrated differential temporal responses to mIA in fetus-matched placental and yolk sac tissues [43], making this an important area of study for future research efforts, but within the scope of this review, we focus on placental effects.

Infectious pathogens such as influenza, as well as pathogen-associated molecular patterns (PAMPs) such as lipopolysaccharide (LPS), a TLR4 agonist to mimic bacterial infection, or viral mimetics such as polyinosinic:polycytidylic acid (poly(I:C)), a double-stranded RNA analogue and TLR3 agonist, have been widely applied to simulate infection and hence maternal immune function responses in mIA models [30,31,42,60]. These treatments lead to signal transduction and downstream activation of transcription factors such as nuclear factor kappa β (NF-κβ) with consequent transcription of pro-inflammatory and anti-inflammatory mediators such as cytokines and chemokines as well as complement proteins [31], with acute phase elevations of these detected systemically in maternal plasma [43,61–66]. These models are used widely in the context of NDDs such as SZ or ASD, in line with the epidemiological observations following infection [26,27]. However, as already noted, these models do differ in pathogen, dose and gestational timing of immunostimulation [15,40,42,60], as well as offspring phenotype being investigated, generating complexity as regards outcomes reported across manipulations [31,44].

In particular, the gestational timing of immunostimulant administration and magnitude and endurance of mIA responses are likely to determine which neurodevelopmental characteristics are affected, dependent on developmental stage, while governing the scope of apparent neurological deficits in the offspring [11,30,31,44]. Another source of experimental variability worth commenting on is the variable molecular weight within some supplier sources of poly(I:C), together with evidence of endotoxin contamination, resulting in different maternal and fetal outcomes [61,67]. Model animal supplier can also affect offspring outcomes, with C57BL/6J mice from specific suppliers showing mIA-induced deficits in prepulse inhibition (PPI) of startle, suggesting variability even within the same strain and an influence of rearing environment [68]. Furthermore, factors inherent to preclinical modelling may also attenuate (or exaggerate) offspring phenotypic display. For example, housing conditions and environmental enrichment have been shown to offset mIA-induced cognitive deficits in both male and female offspring [69,70], suggesting that different postnatal outcomes may be invoked through a degree of neural plasticity related to the offspring’s environmental experience. Such sources of variability can be capitalised on to reflect clinical mIA in different contexts [71].

Despite these complexities, the translational validity of these models relies on behaviour in model species that mimics the phenotypes of NDDs, such as changes to social functioning [72,73]; endophenotypes such as sensorimotor gating dysfunction as measured by impaired PPI of startle [74,75]; or cognitive tasks reflecting analogous processes between the model species and humans [76]. Their face validity is supported by disease-related phenotypes including cognitive deficits of specific relevance to NDDs, such as deficits in the attentional set shifting task [77]. This task is designed to reflect the same prefrontal mechanisms of the human intradimensional/extradimensional shift test or Wisconsin Card Sorting Task, procedures used to measure executive functioning in patients across the spectrum of NDDs [76,78]. Further, deficits in the novel object recognition task, a task often with high predictive and face validity to deficits seen in SZ [79] can be seen following mIA [33,80]. Additionally, diagnosis-specific phenotypes can be measured through specific tasks or attention to specific behaviours. Deficits in social preference have been seen following mIA [81–83] and can be used to reflect social withdrawal or negative symptoms of SZ [73], whereas the scoring of typical behaviours such as autogrooming could be used to reflect repetitive behaviour as found in ASD [84]. Endophenotypes such as PPI of the startle response are affected by mIA, mirroring the impairment seen in several neuropsychiatric disorders [85–87], further supporting the predictive and face validity of mIA model use. The putative mechanisms that link mIA-induced changes to offspring NDD susceptibility will be discussed further in the sections below.

## mIA-induced cytokines as primary mediators of neurodevelopmental disorder; effects at the placental interface

Against the backdrop of raised maternal cytokines and chemokines induced by mIA in response to maternal infection during pregnancy, there has been considerable research interest in trying to delineate the mechanisms by which such maternal immunomodulation influences fetal neurodevelopment to ‘program’ NDDs in the offspring [22]. There is a well-established appreciation that metabolic and physiological perturbations in the maternal environment are ‘sensed’ by various signalling mechanisms in the placenta, eliciting changes downstream in placental function, that serve to adapt to the altered maternal environment. This can result in disturbed fetal homeostatic balance, with the potential to influence fetal growth and development [88–90]. The gestational timing of exposure to ‘stressors’ will be an important determinant of changes invoked in placental development and function and the downstream impacts and subsequent developmental programming of the fetus [90].

Disturbed maternal cytokine balance, such as that evoked by mIA, has received considerable attention as cytokines function in the development of the placenta, as well as the immune and nervous systems and importantly, this involves a range of pro-inflammatory and anti-inflammatory cytokines [91–95]. Hence, cytokines are not only modulators of immune activity, but can also be regarded as critical autocrine and paracrine mediators that modulate signalling between distinct non-immune tissues to regulate a diverse array of functions through the activation of various signal transduction cascades [91,94]. In this way, cytokine induction in response to maternal infection can adversely affect neurodevelopment [94], discussed further in the next section.

This environmental setting also leads us to consider whether stimulated maternal cytokine production arising from mIA results in cytokines crossing the placenta or affects placental function, especially functions such as placental nutrient uptake and fetal nutrient provision, as these have the potential to drive altered fetal brain development, whilst also providing precursors essential for neuromodulatory regulation [43,96–98]. Three possible scenarios seem plausible: (i) raised maternal cytokines cross the placenta to evoke changes in fetal development, (ii) raised maternal cytokines directly affect placental function with downstream fetal developmental impacts, and/or (iii) placental production of cytokines invokes signal transduction events that elicit changes in placental development and adaptation of placental function, with consequences for fetal development. We will now consider each of these possibilities, but they may not be mutually exclusive in eliciting effects at convergent functional loci.

### Do maternal cytokines cross the placenta?

To affect placental function, or indeed be able to cross the placenta, routes of influx of maternal cytokines into placental tissue must exist. Cytokines are postulated to enter and cross the placenta by simple diffusion through paracellular trans-trophoblastic water-filled channels, dependent on the permeability characteristics of the placenta [99], which may change over the course of pregnancy [100]. Indeed, fetal transfer of [^125^I]IL-6 was significantly greater at mid-gestation than at late gestation in pregnant rats [99]. Yet, maternal [^125^I]IL-1β showed minimal fetal transfer across rat placenta [101]. Maternal administration of [^125^I]IL-2 to pregnant mice dams at mid-gestation demonstrated significant accumulation of the tracer in the fetuses, placenta and amniotic fluid, implicating transplacental transfer of IL-2 [102]. Recent studies in human placenta have identified trans-syncytial nanopores that connect directly the maternal- and fetal-facing sides of the syncytial epithelium of human placenta, the syncytiotrophoblast, which may provide a potential pathway for paracellular diffusion [103] and the passage of cytokines between the mother and her developing fetus. Therefore, these observations provide a putative transplacental route by which maternal cytokines can enter the fetal circulation [99,102] and potentially cross the fetal blood–brain barrier [104], which may also raise barrier permeability through changes to endothelial cell junction integrity and increased astrocyte damage [105,106], modifications likely to permit direct effects on fetal neurodevelopment. In this context, it is worth noting that mIA induced by LPS or poly(I:C) in rodent models has been reported to evoke relatively rapid (within a few hours) changes in the transcription of genes encoding pro-inflammatory cytokines such as IL-6, TNFα and IL-1β in the fetal brain ([44]; Table 1). Further evidence in support of the transplacental passage of maternal cytokines comes from studies showing that maternal [^125^I]TGF-β1 in pregnant mice was detected in placental tissue and rapidly crossed the placenta to the fetus where it could be recovered from various fetal tissues such as liver, lung, bone and muscle in a time-dependent manner [107]. However, others have shown that in pregnant rat dams while maternally administered [^125^I]TNFα is sequestered by the placenta, transfer to the fetus is negligible [108]. Other models have yielded inconsistent observations; for example, using the *in vitro* perfused human placenta model, IL-8 was not transferred [109], contrasting with IL-6 which demonstrated bidirectional transfer across the tissue [110], while others using this model have failed to find evidence of IL-6, TNFα, IL-1β or IL-1α transfer [110,111].

**Table 1:** Changes in expression of inflammatory factors in fetal brain, placenta and other fetal compartments in response to mIA

Fetal brain	Placenta	Other fetal compartment(s)	Immunogen and dose (per kg bodyweight or dam)	Gestational day (GD) of administration	Species	Reference
↑IL-6 (6 h)			Poly(I:C) 2.5 or 5 mg/kg i.p.	GD9	Mouse	[342]
	↑ IL-6, IL-12p40, MCP-1 (CCL2), MIP-1β, RANTES (CCL5), KC (CXCL1) (2 h, 4 h) ↑IL-1β, IFNγ, MIP-1α (4 h)		Poly(I:C) 4.5 mg/kg i.p.	GD16.5	Mouse	[133]
↓ IL-1β (3 h), ↓ IL-10 (3 h), ↑IL-6 (3 h, 6 h), ↑IL-1β (6 h), ↓ mRNA for IL-1β (3 h), IL-10 (6 h)			Poly(I:C) 5 mg/kg i.v.	GD9	Mouse	[343]
↑IL-1β, IL-10 (3 h), ↑IL-6 (6 h), ↓ mRNA for IL-1β, TNFα (3 h), ↑mRNA for IL-6, IL-10, TNFα (6 h)			Poly(I:C) 5 mg/kg i.v.	GD17	Mouse	[343]
↑IL-1β, IL-5, IL-4 (6 h)			Poly(I:C) 5 mg/kg i.p.	GD9	Rat	[344]
↑IL-6 (3 h, GD12.5)			Poly(I:C) 5 mg/kg i.v.	GD12.5 GD17.5	Mouse	[281]
HMW: ↑IL-6, TNFα, CXCL1 (3 h) LMW: ↑IL-6, TNFα, CXCL1 (3 h)	HMW: ↑IL-1β, IL-6, IL-10, IL-17a, TNFα, CXCL1, MCP-1 (3 h) LMW: ↑IL-6, CXCL1, MCP-1 (3 h)		Poly(I:C) 5 mg/kg i.v. (HMW and LMW)	GD12	Mouse	[61]
↑mRNA for IL-6 (6 h)	↑mRNA for IL-6, TNFα (6 h, 48 h)		Poly(I:C) 10 mg/kg i.p.	GD14	Rat	[96]
	↑mRNA for IL-6 (3h), TNFα (3 h, 24 h), ↓mRNA for IL-1β (24 h)	↑mRNA for TNFα (3 h, 24 h), IL-6 (24 h) in visceral yolk sac	Poly(I:C) 10 mg/kg i.p.	GD15	Rat	[43]
↓ MIP-1α (6 h), ↑MIP-1α (24 h), ↑IP-10 (CXCL10) (6 h, 24 h), ↑ IL-7 (6 h), ↑IL-13, (6 h), ↑IL-1β (24 h), ↑MCP-1 (24 h)			Poly(I:C) 20 mg/kg i.p.	GD16	Mouse	[345]
↑CXCL10, CCL5 (6 h)	↑CXCL10, CXCL12 (6 h)		Poly(I:C) 20 mg/kg s.c.	GD12.5	Mouse	[346]
↑IL-12p40 (48 h)	↑IL-6, TNFα, IL-12p40 (48 h)		Poly(I:C) 20 mg/kg i.p.	GD12	Mouse	[347]
	↑TNFα (2 h)	↑ TNFα in liver/spleen (2 h)	Poly(I:C) 10 or 20 mg/kg i.p.	GD16	Rat	[348]
↑mRNA for IL-6 (3 h)	↑IL-6 (3 h, 6 h, 24 h) ↑mRNA for IL-6, TNFα (3 h)		Poly(I:C) 20mg/kg i.p.	GD12.5	Mouse	[134,138]
↑mRNA for IL-1β (6 h), IL-6 (2 h), MCP-1 (2 h, 6 h)			LPS 50 μg i.p.	GD18	Mouse	[349]
↑mRNA TNFα, IL-1β (6 h)	↑mRNA for IL-1β, IL-6, TNFα (6 h)		LPS 50 μg/dam	GD15	Mouse	[330]
↑mRNA TNFα, IL-1β (6 h)	↑mRNA for IL-1β, IL-6, TNFα (6 h)	↑IL-6 in amniotic fluid (6 h)	LPS 50 μg/dam	GD18.5	Mouse	[350]
	↑ TNFα (2 h), IL-1β (4 h, 8 h), IL-6 (8 h)	↑IL-1β in fetal plasma (4 h)	LPS 50 μg/kg i.p.	GD18	Rat	[66]
↑ TNFα, MCP-1 (CCL2), IL-6, NFκβ (4 h)			LPS 100 μg/kg i.p.	GD18	Rat	[351]
	↑mRNA for IL-1β (12 h), IL-6 (6 h, 12 h), TNFα (1 h, 6 h, 12 h)	↑IL-1β (1 h, 6 h, 12 h) IL-6 (6 h, 12 h), TNFα (1 h, 6 h) in amniotic fluid	LPS 100 μg/kg i.p.	GD18	Rat	[64]
↑IL-6 (3 h)			LPS 120 μg/kg i.p.	GD17	Mouse	[352]
↑IL-1β, IL-2, IL-5, IL-18 (6 h), ↑IL-6, ↓IL-4, IL-18 (12 h), ↑IL-1β, IL-6, TNFα, IL-2, IL-4, IL-5, IL-7, IL-10, IL-18 (24 h)			LPS 250 μg/kg i.p.	GD15	Rat	[353]
↑mRNA for IL-1β (1 h, 4 h, 24 h, 48 h), ↑mRNA for TNFα (1 h, 4 h, 24 h), ↑mRNA for iNOS (24 h, 48 h)	↑mRNA for IL-1β, TNFα, IL-6 (4 h, 24 h, 48 h) ↑mRNA for iNOS, MCP-1 (CCL2) (24 h, 48 h)	↑IL-6, TNFα in amniotic fluid (4 h, 24 h)	LPS 500 μg or 1 mg/kg i.p.	GD18	Rat	[354,355]
	↑IL-6, TNFα, IL-1β (2 h, 8 h)	↑IL-6 in amniotic fluid (2 h, 8 h)	LPS 500 μg/kg i.p.	GD16	Rat	[356]
↑TNFα (1.5 h)		↑TNFα in liver and amniotic fluid (1.5 h)	LPS 500 μg/kg i.p.	GD17	Mouse	[357]
↓TNFα (2 h)	↑IL-6, TNFα (2 h)	↑TNFα in amniotic fluid (2 h)	LPS 2.5 mg/kg i.p.	GD16	Rat	[356]
	↑IL-6, TNFα (6 h) at GD16, ↑TNFα (6 h) at GD20		LPS 1 or 10 mg/kg for 6 h	GD16 GD20	Rat placental explant (secretion)	[358]

**Abbreviations:** CCL, C-C motif chemokine ligand; CXCL, C-X-C motif chemokine ligand; HMW, high molecular weight; IP, interferon gamma-induced protein; i.v., intravenous; i.p., intraperitoneal; IL, interleukin; iNOS, inducible nitric oxide synthase; LMW, low molecular weight; MIP, macrophage inflammatory protein; NF-κβ, nuclear factor kappa β; MCP-1, monocyte chemoattractant protein-1; s.c., subcutaneous; TNFα, tumour necrosis factor α; ↑ or ↓ indicates increased or decreased expression respectively.

### Do maternal cytokines directly affect placental function?

Irrespective of whether cytokines are transferred across the placenta, *in vitro* studies support direct effects of exogenous cytokines on placental nutrient transport function. For example, TNFα and IL-6 stimulate system A-mediated amino acid transporter activity in cytotrophoblast cells isolated from human placenta [112,113], through a MAPK-dependent pathway [112] and increased phosphorylation of signal transducer and activator of transcription 3 (STAT3) [113], respectively. Yet, neither IL-6 nor TNFα at comparable doses alter system L-mediated leucine uptake in the same cell type [113], showing specificity of action. Consistent with this notion, LPS and poly(I:C) stimulate system A activity, but do not affect system L activity in the same cell type [114]. In pregnant rat dams, an acute maternal injection of TNFα significantly reduced placental transport of the leucine analogue [^14^C]cycloleucine [115]. In primary placental cell cultures from mice, treatment with TNFα decreased *Slc38a2* mRNA expression, which encodes SNAT2 transporter subtype of system A, but this effect was not observed with IL-1β [116], again highlighting the specificity of cytokine effects. Cultured human placental explants treated with IL-17A, IL-22 or IL-23, alone or in combination, altered the gene expression for candidate drug transporters in a selective manner [117]. Furthermore, the placenta expresses an array of cytokine receptors and interaction with these can induce a variety of signalling pathways that may alter placental growth and differentiation [92], thereby also affecting function. Indeed, we have recently shown that mIA-induction by poly(I:C) in a rat model results in reduced placental weight, increased fetal/placental weight ratio, and altered transplacental leucine transport [43].

This latter observation is of particular interest as system L activity, which mediates the placental transport of leucine, also transports the amino acid tryptophan [118], the precursor for serotonin (5-hydroxytryptamine) synthesis [98,119]. This mechanism has developmental importance as some of the maternal tryptophan taken up by the placenta is converted to serotonin by placental tryptophan hydroxylase (TPH1) activity, providing an exogenous source of serotonin for fetal neurodevelopment [98,119]. In mice, placental serotonin synthesis capacity is greater in the earlier gestational period, with the converse apparent in fetal brain [119], revealing a progressive developmental switch from an early dominant placental exogenous source of serotonin to a later endogenous source synthesized by the brain [120]. The human placenta also demonstrates serotonin synthesizing capacity in the first trimester, highlighting the importance of this placental source of serotonin for fetal neurodevelopment across species [119]. Further, some elegant placental perfusion experiments demonstrated that placental-synthesized serotonin can be released into the fetal circulation, confirming this pathway is crucial to maintain normal levels of serotonin for early fetal forebrain development over a time period coincident with cortical neurogenesis, migration and initial axon targeting [119,120]. Following poly(I:C) induction of mIA and raised maternal IL-6, placental TPH1 activity was significantly increased, concomitant with significantly increased fetal forebrain serotonin concentration, and suppression of serotonergic axon outgrowths within the fetal forebrain [98]. Importantly, placental production and release of serotonin was significantly greater in the poly(I:C)-treated group as compared to saline-treated controls [98], providing a mechanism whereby maternal inflammation during pregnancy can influence fetal brain development through placental-mediated changes in serotonin-synthesizing function. Taken together, these observations implicate direct effects of cytokines on placental function.

### Does placental endogenous production of cytokines affect placental function?

It is important to note that within the setting of mIA, vertical transmission of pathogen across the placenta is not a prerequisite for altered placental cytokine concentration. For example, both human and animal studies have shown that the influenza virus, or indeed antibodies produced to it, do not cross the placenta nor are found in the brains of offspring of exposed mothers [121–123]. While induction of mIA using infective pathogens such as influenza have shown that placental development is impaired, with an increased number of inflammatory cells within the placenta, as well as altered expression of placental genes associated with NDDs, inflammation and immune response [122]. Delineation of the inflammatory and cytokine effects at the locus of the placental/fetal interface have been greatly aided by the widely applied use of non-infective mimetics such as LPS and poly(I:C) in rodent models to induce mIA (which, as already discussed, have good model validity). From such studies, there is evidence of rapidly altered cytokine concentrations apparent in various fetal compartments including placenta, amniotic fluid and fetal brain ([31]; Table 1). It is worth emphasizing that across the maternal-fetal interface, the magnitude and direction of cytokine (and chemokine) changes induced by mIA in maternal plasma, is not necessarily mirrored by that seen in the placenta or fetal brain [61], which may argue for local regulation of fetal endogenous cytokine synthesis, with the potential to affect neurodevelopmental events and increase NDD risk [44].

In the aforementioned rodent models, LPS and poly(I:C) are used as ligands for TLR4 and the endosomal TLR3, respectively [124]. Human and rodent placentas express both of these receptors [125–131], from early in pregnancy, along with other TLRs [126]. LPS- or poly(I:C)-mediated mIA induction in rodent models leads to up-regulated placental TLR4 or TLR3 placental expression respectively [128–130], stimulated placental cytokine gene expression, increased placental cytokine concentration, and altered fetal brain cytokine gene expression and concentration (Table 1), together with altered placental expression of different classes of amino acid transporters and their activity [43,96,97]. Collectively, together with other placental changes, these are likely to lead to a disturbed fetal physiological milieu, and the genesis of neurodevelopmental changes with impacts on NDD risk susceptibility [43,44,132].

Although the placenta has the capacity to synthesize a wide variety of cytokines [91,133], some elegant studies by Hsiao and Patterson [134] demonstrated that when the ability of placental trophoblast cells (i.e. fetal cells) to produce IL-6 was abolished by *Il6* gene ablation in mice, the acute rise in placental IL-6 concentration following mIA induction by poly(I:C) (Table 1) persisted; hence this was not contributed to by fetal trophoblast cell synthesis, but rather by maternal-derived IL-6 produced from activated maternal immune cells within the placenta. This inference was also confirmed by the negligible placental IL-6 response in *Il6* null-dams [134]. The increase in placental IL-6 concentration led to the activation of STAT3 signalling pathway within the spongiotrophoblast cells of the placental JZ, resulting in reduced expression of placental-specific hormones such as insulin-like growth factor 1 (IGF-1) and growth hormone (GH) [134] that regulate placental development, placental nutrient transporter activity and fetal growth, along with nutrient partitioning to the fetus [135–137]. While IL-6 appears to be a key player in the etiopathology of NDDs, as shown by a lack of behavioural deficits seen in poly(I:C)-treated *Il6* knockout mice, or when endogenous IL-6 activity is blocked by neutralization with a targeted antibody [86], it is likely that there is a more complex, temporal-dependent, and integrative influence of multiple cytokines induced by the mIA response (Table 1). For instance, we have recently shown that the offspring of poly (I:C)-treated dams showed early behavioural and adult cognitive deficits that were correlated to the maternal systemic TNFα response [77]. That said, many mIA-associated transcriptional changes found in the offspring’s brain necessitate maternal IL-6 synthesising capacity [138] and are largely normalised by IL-6 targeting antibody [86], indicating a key dependence on maternal IL-6, which might be subsequent to the temporality of TNFα and IL-1β elicited effects [66]. Furthermore, there is also a reliance on signalling through the placental trophoblast receptor for IL-6 (IL-6Rα), with mIA-induced inflammatory signalling responses through the STAT3 pathway and expression of inflammatory genes in fetal brain dependent on its activation, along with the manifestation of behavioural abnormalities in the offspring [138].

### Placental programming of neurodevelopmental disorders

Hence, together these studies reveal that the elicited maternal mIA response undergoes signal transduction at the locus of fetal trophoblast cells within the placenta to evoke paracrine effects that modulate trophoblast cell function within the placenta. This leads to downstream activation of placental signalling pathways with impacts on placental function, causing perturbed fetal homeostasis. This, in turn, causes activation of several signalling cascades within the fetal compartment that ‘prime’ for dysregulated fetal immune function and the induction of aberrant fetal brain signalling events, leading to altered fetal neurodevelopmental trajectory and ensuing developmental programming of behavioural deficits in the offspring. In this way, the mechanistic linkage pathway can be viewed as stimulated maternal pro-inflammatory cytokines (‘sensors’) in response to environmental insults (infection), leads to associated increases in pro-inflammatory cytokines in the placenta and fetal brain (‘transducers’), resulting in altered fetal behavioural outcomes (‘effectors’) [139].

While such a scheme is likely to be somewhat of an oversimplification of the convergence of molecular events that drive NDDs, it places placental responses to mIA as a key mediator of mechanistic importance in the etiopathology of NDDs. Hence, the ‘placental programming of NDDs’ paradigm has gained widening appreciation. In this framework, various maternal environmental stressors, including maternal inflammation, converge on neurologic disease through the integrated placental response to a diverse range of maternal signals that evoke adaptive/maladaptive changes in both the placenta and the developing brain, modulated by epigenetic changes [44,139–142]. Indeed, a variety of maternal stressors that evoke a stimulated maternal immunogenic response produce histological, transcriptional and behavioural manifestations in the offspring brain that are similar to those seen in LPS and poly(I:C) mIA models, implying a degree of convergence at the level of mediating molecular mechanisms [139]. In this context it is interesting to note that others have drawn attention to the notion that critical developmental processes in the placenta and fetal brain appear to be governed by the same environmental cues, emphasizing the close regulatory ’cross-talk’ between these two tissues, with such developmental synchronization disrupted by maternal metabolic stress and the adaptations invoked by the placenta [143]. It is also noteworthy that a group of SZ risk-associated genes are highly expressed in the placenta during early life and are modulated by biological stress, leading to the notion that genetic variants associated with SZ are convergent on a developmental trajectory that affects placental responses to stress [144].

In the subsequent sections, we elaborate further on the mechanisms of cytokine-mediated modulation of neurodevelopment and relate findings to neurodevelopmental outcomes in the context of the recent SARS-CoV2 pandemic in the final section.

## Cytokines and inflammation in neurodevelopmental disorders

### Cytokines in brain development

Normal brain development begins early post-conception and continues into early adulthood, comprising various processes organised both spatially and temporally [145,146]. Early stages of brain development occur with neural tube convergence and patterning and proliferation of neuroepithelial cells which transition to radial glial cells, the essential neural progenitor cells (NPCs). Proliferation and differentiation of NPCs into neurons (neurogenesis) then occurs in the ventricular zone (VZ) and subventricular zone (SVZ). Neurogenesis is followed by gliogenesis, the production of astrocytes and oligodendrocytes. Finally, myelination, synaptic sculpting and modulation of circuitry networks occur in the postnatal period [145–147]. This creates a relatively large temporal window during which the brain is vulnerable to developmental perturbations [148]. As already alluded to, prenatal and early life insults, notably mIA and placental dysfunction, are thought to induce inflammatory signalling pathways, in particular cytokine signalling pathways, in the fetal brain which are hypothesised to disturb normal neurodevelopmental trajectories and predispose individuals to neuropathology [149–152]. This is underscored by the properties of cytokines acting as critical signalling molecules during normal brain development [153]. Early work showed that cytokines are present in the early fetal brain and that their concentrations corresponded with critical developmental stages [154]. Subsequently, cytokines were shown to regulate various neurodevelopmental processes, and that disturbances in the normal temporal pattern of brain cytokines profiles have the potential to cause dysregulation of these processes and thus disturb neurodevelopment both prenatally and during postnatal brain development [155–157]. In line with this hypothesis, several studies have evaluated changes in cytokine expression and neuroinflammatory markers in the developing brain of offspring following mIA and reported ongoing elevations in pro-inflammatory cytokine (notably, IL-6, TNFα and IL-1β) expression in both the fetal and postnatal brain ([44,62]; Table 1). Of note, persistent neuroinflammation has also been identified in the brains of individuals with NDDs [158,159] lending weight to the role of these signalling molecules in the pathology of NDDs.

#### Neurogenesis and neuronal migration

Neurogenesis is the process of proliferation and differentiation of NPCs to give rise to neurons [160]. These newly generated neurons then migrate outwards into the cortical layers. *In vivo* perturbation of neurogenesis and neuronal migration leads to various behavioural patterns analogous to those observed in NDDs [161,162]. Neurogenesis has been shown to be both increased and inhibited by individual cytokine signalling pathways [156]. Cytokine imbalances as a result of prenatal insults, notably changes in the IL-6 family of neuropoietic cytokines, could therefore disturb the NPC proliferation rates and the temporal regulation of neurogenesis, and in turn disturb the normal neuronal pools within the cortical layers [153,156,163]. Further, cytokines, notably pro-inflammatory IL-1β, and chemokines have also been shown to influence neuronal migration [94,164]. Notably, chemokines have been demonstrated to act as a chemoattractant within the cortical surface layers, working to regulate the positional migration of neurons. As with neurogenesis, *in vivo* inhibition of these signalling pathways has been shown to cause cortical dysregulation and mis-patterning [94,165,166]. These disturbances are likely to contribute to incorrect neuronal ratios and signalling in the postnatal and developing brain.

#### Gliogenic switch

Following neurogenesis there is a temporal switch to gliogenesis, the production of first astrocytes and then oligodendrocytes from the NPC pool. These sequential fate switches are dependent on key signalling pathways and SVZ patterning [145,146]. Similar to neurogenesis, gliogenic switches are regulated by the IL-6 family of neuropoietic cytokines through the temporal induction of distinct astroglial and oligodendroglial progenitor cell markers in the SVZ NPC pool [167,168]. Hence, pro-inflammatory signalling within the fetal brain, particularly IL-6, has the potential to dysregulate these temporal cell fate decisions by promoting an early gliogenic switch, thereby reducing the neuronal pool. Indeed, abnormal gliogenesis and glial cell ratios have been shown to be risk factors for NDDs [169,170].

#### Oligodendrocyte maturation and myelin plasticity

Oligodendrocytes are the primary myelinating cells in the brain that arise from oligodendrocyte progenitor cells (OPCs) around the time of birth [171]. These cells are responsible for axon ensheathment and formation of the major white matter tracts. Critically, OPCs persist throughout life and maintain the capacity to differentiate in mature oligodendrocytes. This progenitor pool therefore aids in neural plasticity and the need for remyelination through development. Proinflammatory cytokines have been shown to inhibit OPC proliferation and survival and hence reduce myelin plasticity in the developing brain [172,173]. Myelin plasticity is critical for normal neuronal signalling and protection of neurons from damage in later-life, with disturbed myelin plasticity present in various NDDs [174]. Further, neuroinflammation can cause damage to myelin sheaths, leading to demyelination along with axon and synaptic damage, preventing normal neuronal signalling [175].

### Microglia and astrocyte activation and their role in neuroinflammation

Cytokine expression in both the prenatal and postnatal brain is primarily isolated to microglia and astrocytes; hence these cells are thought to be critical in mediating the outcomes of any downstream output of cytokines and inflammatory signalling [153,176].

Microglia represent the resident immune cells of the brain responsible for mediating inflammatory processes and arise from yolk sac progenitor cells in early development [177,178]. Inflammation and stress activate microglial cells, promoting the secretion of cytokines to induce a neuroinflammatory phenotype [179,180]. Activation of microglia and the stimulated production of neuroinflammatory cytokines has a negative impact on the expression of neurotrophins such brain-derived neurotrophic factor (BDNF) [181,182]. BDNF plays a key role in neurodevelopment [183,184], being essential for learning, cognitive and memory functions [185]. Microglial-derived BDNF influences synaptic plasticity [186] and a reduced expression of BDNF associates with altered behaviours and cognitive deficits [182,187]. Offspring of mIA-affected mothers have been shown to exhibit reduced BDNF expression [188–190], which may link to altered behaviours observed in adulthood [189,190]. BDNF downstream signalling may also influence the expression of various synaptic protein targets, thereby affecting synaptic function [184]. Microglia are also responsible for the phagocytosis of cell and myelin debris and synapses, a process critical for normal synaptic connectivity during development [178,191]. Hence, excess microglial activity as a result of inflammation can lead to excess synaptic cleavage and neuronal damage as well as perpetuating ongoing neuroinflammation leading to altered function.

Astrocytes represent a diverse group of cells within the brain, and they function in synapse formation, neurotransmission and metabolism throughout development [192]. Astrocytes are also susceptible to activation by cytokine signalling and active microglia leading to reactive astrogliosis [193]. This is accompanied by a functional change during which astrocytes begin to proliferate and secrete pro-inflammatory mediators which can cause damage and synaptic loss, with a seemingly preferential loss of inhibitory γ-aminobutyric acid (GABA)ergic synapses [194].

In line with their roles in neuroinflammation, increased microglial activity [158,195,196] and astrocyte abnormalities [197,198] have been identified in various NDDs. Moreover, such abnormalities in the developing brain of offspring exposed to mIA were often sex-specific [199–201]. Synaptic loss from microglial and astrocyte activation as a result of neuroinflammation is thought to contribute to the excitatory/inhibitory (E/I) imbalance [194] frequently reported in NDDs, thereby contributing to behavioural abnormalities [202,203].

### Neuronal signalling and neuroinflammation

As already outlined, neurodevelopment is a highly regulated process with precise spatial and temporal benchmarks with prenatal and postnatal neuroinflammation causing various neuronal signalling dysfunctions. Analysis of gene expression after infection with influenza, poly(I:C) or IL-6 injection in early neurodevelopment in rodent models indicated common patterns of change in gene expression relevant to neurogenesis and neuroprotection in the acute phase of infection [204]. Indeed, there are many mIA studies with evidence of decreased neurogenesis or cell survival in fetal, juvenile and adult brain (Table 2). Interestingly, as shown in Table 3, markers of cell death appear absent in animals exposed to mIA in early development (gestational day (GD)8-13.5) but present in offspring exposed in late gestation (GD15-20) in both prenatal and postnatal tissue, which again serves to highlight the developmental dependency of neurodevelopmental responses arising from mIA exposure.

**Table 2:** The effect of mIA on neurogenesis and cell survival in prenatal and postnatal brains

Gestational day	mIA induction method	Region investigated	Age of offspring measurement	Measure	Change	Reference
**Transgenic overexpression of IL-6**	IL-6	DG	PD84-112	24 h after BrdU	↓ granule cell layer	[359]
			PD115-143	31 days after BrdU NeuN	↓ granule cell layer ↓ BrdU/NeuN in granule cell layer	
**8, 10 + 12**	LPS	CA1	PD84	Cell number	↓	[360]
			PD280		↓↓	
			PD560		↓↓↓ (age-dependent decrease)	
**8, 10 + 12**	IL-6	CA1	PD168	Nissl stain	↔ in males ↓ in females	[361]
		CA2			↓ in males ↔ in females	
		CA3			↓ in males ↔ in females	
		Hilus			↓ in males ↓ in females	
**9**	Poly(I:C)	DG	PD24	DCX	↓ in subgranular layer and outer granular layer	[343]
**10.5**	LPS	DG	PD22	BrdU on PD21	↓	[362]
				DCX	↓	
			PD91	BrdU on PD90	↓	
				DCX	↓	
**13.5**	LPS	VZ	GD14	pH3 BrdU to dam 2 h post LPS	↓pH3 and BrdU	[363]
			GD15.5		↔pH3 and BrdU	
**14, 16, 18 + 20**	LPS	DG	PD67	BrdU on PD 60-67 NeuN DCX	↓BrdU ↓BrdU/NeuN ↓DCX ↓BrdU/DCX	[364]
			PD97		↓BrdU ↓BrdU/NeuN	
**15 + 16**	LPS	DG	PD23	BrdU 4 h after last LPS injection	↔	[365]
			PD14	BrdU 2 h before cull	↓	
			PD42	BrdU on PD14	↓	
			PD60	BrdU 2 h before cull	↔	
			PD88	BrdU on PD60	↔	
**15**	LPS	Cortex	GD15 (+4 hours after LPS)	mRNA /pathway analysis	↓mRNA for genes relevant to neurogenesis	[228]
**15**	Poly(I:C)	DG	PD196	BrdU (PD unknown)	↓	[232]
**15**	Poly(I:C)	DG	PD37	BrdU on PD 14 16	↓	[231]
			PD57	BrdU on PD 34 36	↓	
			PD72	BrdU on PD 49 51	↔	
			PD100	BrdU on PD 77 79	↔	
**15**	Poly(I:C)	DG	PD90	BrdU (PD57-59) Ki67 Nestin DCX NeuN	↓ Ki67 ↓ Ki67/DCX ↔ Ki67/Nestin ↓ BrdU ↓ BrdU/DCX ↔ BrdU/Nestin ↓BrdU/NeuN	[366]
**16, 18 + 20**	IL-6	CA1	PD168	Nissl stain	↔ in males ↓ in females	[361]
		CA2			↓ in males ↔ in females	
		CA3			↓ in males ↓ in females	
		Hilus			↓ in males ↓ in females	
**16**	Poly(I:C)	Cortex	GD18	BrdU on GD16	↓	[367]
**17**	Poly(I:C)	DG	PD24	DCX	↓ outer granular layer	[343]
**18**	Poly(I:C)	DG	PD28	BrdU on PD 27-28 DCX	↓ BrdU ↓BrdU/DCX ↓DCX	[368]
**18 + 19**	LPS	DG	PD26	BrdU 4 h after last LPS injection	↓	[365]
			PD14	BrdU 2 h before cull	↔	
			PD42	BrdU on PD14	↓	
			PD60	BrdU 2 h before cull	↔	
			PD88	BrdU on PD60	↔	

**Abbreviations:** BrdU, bromodeoxyuridine; CA1, CA1 area of the hippocampus; CA2, CA2 area of the hippocampus; CA3, CA3 area of the hippocampus; DCX, doublecortin; DG, dentate gyrus; GD, gestational day; IL-6, interleukin-6; LPS, lipopolysaccharide; NeuN, neuronal nuclei; PD, postnatal day; pH3,phospho-histone H3; VZ, ventricular zone; ↓, ↔, indicates decrease, or no significant change, respectively.

**Table 3:** The effect of mIA on neural cell death

Gestational day	mIA induction method	Region investigated	Age of offspring measurement	Measure	Change	Reference
**8, 10 + 12**	IL-6	Hippocampus	PD28	Caspase-3	↔ in males ↔ in females	[361]
				Procaspase-3	↔ in males ↔ in females	
			PD168	Caspase-3	↔ in males ↔ in females	
**9.0**	Poly(I:C)	DG	PD24	Caspase-3	↔	[343]
**9.5**	Poly(I:C)	S1/M1	GD17.5, PD3, PD6	Caspase-3	↔	[227]
**13.5**	LPS	Cortex	8 h after LPS	TUNEL assay/Caspase -3	↔	[363]
**15**	LPS	Cortex	GD15 (4 h after LPS)	mRNA /pathway analysis	↑ cellular stress and cell death pathways	[228]
**16**	Poly(I:C)	Cortex	GD18	Caspase-3	↑	[367]
**16, 18 + 20**	IL-6	Hippocampus	PD28	Caspase-3	↔ in males ↑ in females	[361]
				Procaspase-3	↔ in males ↑ in females	
			PD168	Caspase-3	↑ in males ↑ in females	
**17**	Poly(I:C)	DG	PD24	Caspase-3	↑	[343]
**19 + 20**	LPS	Subventricular striatal zone	PD1	Caspase-3	↑	[369]
		Periventricular striatum and germinative ventricular zone	PD7		↑	
		Periventricular striatum		TUNEL assay	↑	

**Abbreviations:** DG, dentate gyrus; GD, gestational day; LPS, lipopolysaccharide; M1, motor cortex; PD, postnatal day; S1, somatosensory cortex; TUNEL, terminal deoxynucleotidyl transferase dUTP nick end labelling; ↑, ↔, indicates increase, or no significant change, respectively.

#### Parvalbumin interneurons

The aforementioned neurodevelopmental impacts of mIA and neuroinflammation that result in altered neurogenesis, degradation of extracellular matrices and myelin sheaths, the activation of glial cells and altered cell survival and death, together, constitute disturbances which are likely to influence E/I balance in the developing brain. E/I balance is crucial for healthy brain function, and there is a wealth of evidence that mIA can cause dysregulation of E/I balance [205–209]. GABAergic inhibitory interneurons are critical regulators of this balance, and crucially, several studies have shown impaired function in several NDDs [205–212]. More specifically, parvalbumin interneurons (PVI) are considered critical in maintaining E/I balance [213]. Although important for E/I balance, PVI may be uniquely sensitive to E/I input changes due to the two types of PVI that form during development. Donato et al. [214] identified early- and late-gestational subtypes of PVI, whose plasticity is regulated by excitatory and inhibitory inputs, respectively. Thus, in disorders with reduced excitatory input to early-generated PVI, like SZ, there will be reduced gamma band activity and impaired memory. In disorders with reduced inhibitory input to late-generated PVI, like autism and intellectual disability, there will be impaired plasticity [214]. Therefore, PVIs are sensitive to mIA throughout neurodevelopment and are affected by E/I balance shifts in both directions.

PVI express parvalbumin protein, a calcium buffer first detected in the rodent brain in the second postnatal week, after which its expression steadily increases into adulthood [215–218]. Several studies have investigated the effect of mIA on PVI number or parvalbumin gene expression (Table 4). Considering the outcomes of these studies, it appears early gestation mIA (GD9-12.5) can reduce PVI in juvenile [219,220] and adult offspring [34,220–223]. Although, two studies showed no difference in PVI density [224] or mRNA expression [225] during this period. The data from the next developmental window (GD13-15) is more varied, with some studies showing that mIA decreases parvalbumin mRNA [188], others showing no effect on PVI number [188,226], and a final study seeing an increase in PVI density in the barrel cortex of postnatal day (PD)20 offspring [226]. This variability may be explained by mIA in this gestational period having little effect on proliferation of medial ganglionic eminence-derived neurons, instead altering migration [227–230]. Finally, several studies showed that late gestation exposure to mIA (GD15-19) resulted in decreased PVI cell density, parvalbumin protein and mRNA [222,231–234], while others saw trends only towards decreases [234,235] or no change [236]. The brain region and age studied throughout these studies are inconsistent, so although a firm conclusion cannot be drawn, it is valuable to draw attention to the parallels between the apparent biphasic vulnerability and the biphasic genesis of PVI [214].

**Table 4:** The effect of mIA on parvalbumin levels in the postnatal brain

Gestational day	mIA induction method	Region investigated	Age of offspring measurement	Method of measurement	Change	Reference
**9**	Poly(I:C)	CA1	PD21	IHC	↓	[219]
**9**	Poly(I:C)	Hippocampus	PD70	IHC	↔	[223]
**9-11**	Poly(I:C)	DH	PD168	qPCR	↔	[224]
**10**	Poly(I:C)	Cortex, striatum and hippocampus	Adult	In situ hybridisation (mRNA)	↓ - when combined with PD19 data (*see below)	[222]
**12**	Poly(I:C)	mPFC and amygdala	PD84+	RNAseq	↓ amygdala of offspring with behavioural deficits	[34]
**12.5**	Poly(I:C)	S1	PD70+	IHC	↓ in layers 2+3 only	[221]
**12-17**	Poly(I:C)	mPFC, Hippocampus, NAc	PD78	IHC	↓ PrL and CA1 regions only	[188]
**12-17**	Poly(I:C)	mPFC	PD36	IHC	↓	[220]
			PD79		↓ PrL	
**13-15**	Poly(I:C)	Hippocampus	PD190	IHC	↔ cell number, ↓ intensity	[225]
				qPCR	↓	
**13-15**	Poly(I:C)	DH	PD168	mRNA	↔	[224]
**13.5**	LPS	Barrel cortex	PD20	IHC	↑	[226]
			PD60		↔	
**15**	Poly(I:C)	PrL, amygdala, frontal association cortex	PD21, 35 and 90	IHC	↔	[235]
**15**	Poly(I:C)	DH	PD84	IHC	↔	[236]
**15**	Poly(I:C)	DG	PD112	IHC	↓	[232]
**15**	Poly(I:C)	Hippocampus	PD72 and 100	IHC	↓	[231]
**15-16**	LPS	mPFC, hippocampus, EC	PD90+	IHC	↓mPFC, CA1, lateral EC (males) ↓mPFC (females)	[233]
**17**	Poly(I:C)	Hippocampus	PD133	qPCR	↔	[234]
				WB	↓DH, ↔ VH	
**19**	Poly(I:C)	Cortex, striatum and hippocampus	Adult	mRNA	↓ - when combined with PD10 data (*see above)	[222]

**Abbreviations:** CA1, CA1 area of the hippocampus; DG, dentate gyrus; DH, dorsal hippocampus; EC, entorhinal cortex; IHC, immunohistochemistry; LPS, lipopolysaccharide; mRNA, messenger ribonucleic acid; PD, postnatal day; PrL, prelimbic cortex; qPCR, quantitative polymerase chain reaction; mPFC, medial prefrontal cortex; NAc, nucleus accumbens; RNAseq, next-generation RNA sequencing; S1, sensory cortex; VH, ventral hippocampus; WB, Western blot; ↑,↓, ↔, indicates increase, decrease or no significant change, respectively…

It has also been hypothesised that reduced parvalbumin protein is a compensatory mechanism to adjust to the reduced GABA availability [237]. In keeping with this postulate, several studies have shown a reduction in glutamic acid decarboxylase (GAD)67 or GAD65 enzymes required for GABA synthesis from glutamate [238], in fetal [225,228] and adult tissue [234,236,239–244]. However, some studies in younger offspring (<PD60) showed increases [240,244–246]. Consistent with this conflicting data for GAD, there is evidence of mIA causing increased excitation and reduced inhibition [226,232,239,247–251] and the contrary imbalance [226,250,251]. It is essential to comment on the diversity of regions used to measure these outcomes and to appreciate that excessive inhibition in one brain region may lead to increased excitation in another. The primary finding is the disruption to the E/I balance, which, when intentionally manipulated through the optogenetic stimulation of excitatory neurons or reducing the activity of PVI, can recapitulate the mIA-induced behaviours [221]. Together, these observations implicate a role for dysregulated PVI activity in the etiopathology of NDDs.

## The role of epigenetics in neurodevelopmental disorders

Epigenetics refers to changes in gene functional activity that occur independently to changes in the DNA sequence through DNA modifications such as methylation, histone modifications and post-translational modifications to the histone proteins within the DNA chromatin structure, and non-coding RNA [139]. Together, these epigenetic modifications regulate gene expression changes in response to a perturbed physiological environment induced by various environmental stressors [252]. Of note, changes to the epigenome can be caused by acute as well as longstanding stressor exposure [253]. Hence, the epigenome is both dynamic and labile in nature, and plausibly mediates interactions between the genome and the environment [254], aligning with the concept that epigenetic mechanisms act as mediator between environmental stimuli and bestowed behavioral phenotypes during periods of developmental plasticity [255], although such epigenetic regulation is likely to be highly complex and governed by the severity and duration of exposure and the vulnerability window of developing physiologic systems as well as offspring sex [256].

Within the framework of NDDs, epigenetic programming is gaining focus, with various epigenetic abnormalities identified in the brains of affected individuals [253,257]. Further, prenatal immune activation has been shown to alter the adult neural epigenome in mIA rodent models [44,254]. However, although the mechanisms that promote these epigenetic alterations remain to be fully defined, cytokine signalling pathways are hypothesised to be a key mediator [44,254]. Cytokines can activate enzymes involved in epigenetic mechanisms including DNA methyltransferases (DNMTs) and histone modifiers [159,163,258–261]. As such, altered fetal brain cytokine profiles, as a result of prenatal stress or infection, could lead to cell-specific epigenetic changes which, in turn, may alter the developmental trajectory of individual cell types. Epigenetic regulation therefore provides a likely intermediary mechanism by which inflammatory signalling during brain development can promote long-term developmental consequences and pre-dispose individuals to later life neuropathology. Indeed, during neurogenesis and gliogenesis, the IL-6 family of cytokines can epigenetically inhibit cell linage markers to regulate the temporal formation of neurons, astrocytes and oligodendrocytes [167].

### DNA methylation

Epigenetic regulation involving DNA methylation is essential for normal neurodevelopment [262]. DNA methylation involves the transfer of a methyl group to the cytosine base of DNA by DNMT enzymes. The transcriptional effect depends on the site of methylation, DNA methylation in CpG sites in promoter regions represses gene expression, whereas DNA methylation within the gene body promotes transcription [263]. DNA methylation of regulatory regions of DNA modifies gene expression by keeping DNA in a repressive chromatin state, changing how the regulatory sequence is read by transcription factors, thereby silencing transcription and downregulating gene expression [264]. Altered DNA methylation can lead to enduring effects on gene expression determining phenotypes, while other epigenetic changes, such as histone acetylation, tend to be more transient in nature [262,265]. It also important to note that methylation signatures mark cellular identity within the diverse array of neuronal subtypes within the brain [266].

Studies into the neuro-epigenetic effects of mIA on offspring brain expression demonstrate a role for altered offspring methylation patterns in the development of NDDs, with multiple genes affected, in a development- and brain region-dependent manner, with both hypermethylation and hypomethylation changes observed [44]. The scope of this topic and the individual gene methylation changes seen is too broad to be considered here, but we have recently conducted a systematic review in which we report quantitative changes in gene/protein expression and epigenetic markers, to determine how neurodevelopmental gene expression and epigenetic modulation is altered in the developing brain across different developmental windows from rodent mIA models [44]. The conclusion from our analysis was, importantly, that a set of candidate genes showed reproducible gene expression changes relating to elevated neuroinflammatory markers, reduced myelination and reduced GABAergic signalling [44]. Of note, the majority of genes within the immune/stress response group were cytokines and chemokines. In addition, examination of DNA methylation (and histone modifications) from candidate gene studies showed that epigenetic changes frequently appeared to correlate to corresponding changes in the expression of the targeted genes, suggesting transcriptional ability of specific genes was directly affected by the associated epigenetic changes [44].

### Histone modifications

Histone proteins are subjected to an array of post-translational modifications, including, but not limited to, acetylation, methylation and phosphorylation [267]. Acetylation and methylation modify chromatin via the addition of acetyl or methyl groups, respectively, to lysine (K) or arginine (R) residues on the N-terminal tail of histone proteins [268]. Acetylation neutralizes the positive charge on these amino acid residues which weakens the interconnection between DNA, histones and adjacent histones and thus opens the chromatin structure to allow the transcription machinery to access DNA and activate gene transcription [269,270]. Histone methylation on the other hand, operates by providing binding docks for transcription factors, dependent on amino acid position as well as the state of methylation, which may repress or activate transcription [271]. Consequently, the histone code hypothesis posits that histone modifications have specific biological outcomes depending on the precise combination of histone modifying marks, which can be deciphered by their writers, erasers and readers [272–274]. This complex code of histone modifications not only allows the epigenome to control cellular differentiation and other vital neurodevelopmental processes, but also provides an intermediary mechanism by which adverse environmental conditions can increase the risk of developing a multitude of diseases [272,275].

Hyperacetylation of histone 3 (H3), on a global level, has been reported within the hippocampus of adult mIA offspring, contrasting with reduced global H4 acetylation [276]. While in the juvenile cortex of offspring exposed to poly(I:C) *in utero*, there is hypoacetylation of H3 as determined by reduced H3K9K14ac, and H4 also exhibits hypoacetylation, as shown by reduced H4K8ac, but these changes were not seen in adulthood, nor were they reported in hippocampus, at either juvenile or adult stages [277]. However, H3 and H4 showed significantly increased acetylation at the serotonin transporter gene (*Sert; Slc6a4*) promoter in the hippocampus of mIA mice, which was associated with increased SERT protein expression [276]. Others report that genes implicated in the pathology of SZ, including the glutamate receptor signalling pathway (e.g. *Gria1*), showed decreased promoter-specific histone acetylation (H3K9K14ac) in the juvenile cortex, while in the juvenile hippocampus, there was a relative increase in the hyperacetylation of this histone mark at the same gene loci, with no change in acetylation level of H4K8 at these promoter loci [277]. It is possible that altered acetylation might reflect an altered activity of histone deacetylase, which removes acetyl marks from histones, and whose expression and activity have been reported to be altered in the brain of mIA offspring [276,278]. Acetylation at gene promoters is often highly predictive of gene transcription [279,280] and hence, transcriptomic changes following aberrant acetylation in mIA offspring brains can be correspondingly mapped. Indeed, previous studies selecting genes previously implicated in the pathology of SZ from genetic and/or microarray studies, and postulated to play a role in neurodevelopment, report altered gene expression in the juvenile cortex; hypoacetylation within the promoters was associated with reduced expression of genes such as *Robo1*, *Arhgap18*, *Ntrk3* and *Gria1*, whereas expression of *Disc1*, *Gria1*, *Gria2* and *Ntrk3*, all of which showed hyperacetylation within promoter loci, were elevated within the hippocampus of juvenile mIA offspring [44,277].

In contrast with the changes seen for histone acetylation, the current literature reports no difference in tri-methylation of Lys4 on histone 3 (H3K4me3), which plays critical roles in neurodevelopment [275,281]. However, this does not exclude the involvement of histone methylation in causing brain and behavioural deficits as a result of mIA, as studies using the adult cortex, comprising an extremely heterogenous region, may mask the effect of mIA on histone methylation [220,235]. Also, epigenomic changes may precede distinct changes to the brain phenotype and subsequent behavioural deficits, as supported by aforementioned evidence of global hypoacetylation in the cortex of juvenile (PD24) but not adult (3 month) offspring [277]. Considering that histone modifications are more dynamic than DNA methylation [282], it is surprising that studies have not focused intensively on the fetal brain as a site for altered histone modifiers in the mIA model [163]. This presents an exciting area of investigation for future studies, especially as aberrant histone modifications in human fetal brain are associated with neural disease [283] and histone post-translational modifications are proposed to have lasting effects on the adult brain and behaviour through a prenatal programming axis, that influences susceptibility to NDD risk [284].

Together, the collective observations argue for a complex, integrative network of epigenomic changes that occur in response to mIA, both within the prenatal and postnatal brain, in a brain-region specific manner, and at specific gene loci, with differential patterns of transcriptional regulation. Disturbed epigenetic modulation of genes of relevance for neurodevelopment and function aligns with the concept that mIA-driven epigenetic modulation links to impaired neurodevelopmental patterning and function which programs behavioural phenotypes seen in NDDs, such as deficits in cognitive functions and anxiety-like behaviour [44].

### Non-coding RNAs

Non-coding RNAs (ncRNAs) can be divided into two groups: long ncRNA [285] and short ncRNA [286]. Small ncRNAs can also be subdivided into further groups including short interfering RNA (siRNA), P-element induced wimpy testis-interacting RNAs (piRNA), microRNA (miRNA) and transfer RNA (tRNA)-derived fragments (tRFs) [286,287]. Within these groups, miRNAs are perhaps the most well categorised and most widely expressed in mammalian tissues, comprising nucleotide structures of ∼22bp. They function by binding to complementary mRNA transcripts, regulating gene expression by transcript and translation interference, usually by promoting transcript cleavage/degradation [286,288]. Studies evaluating miRNA function in human tissues have estimated that >30% of the total human transcriptome is regulated by miRNAs [289–291]. NcRNAs has important roles in normal brain development and function. The brain shows distinctly high levels of ncRNA expression compared to any other tissue [292]. Indeed, profiling of mice brain miRNA expression across development has suggested that 20% of miRNAs undergo significant changes of expression (both down and up regulation) over the developmental time-course [293]. Moreover, specific roles for ncRNA in brain development and function are emerging. Deletion of the long ncRNA, *Evf2*, in mice reduces hippocampal GABAergic interneuron density and restricts forebrain development [294], while loss of function of specific miRNAs, including miR-219 [295], miR-9 [296,297] and miR-124 [298,299] have been shown to inhibit neurogenesis and gliogenesis.

Taken together, these studies suggest an important role for ncRNA in brain development, that may be dysregulated in NDDs. That said, ncRNA is perhaps the least studied epigenetic mechanism evaluated in mIA models. Global miRNA expression analysis in the cortex of prenatal poly(I:C)-exposed offspring found major differences in miRNA expression, with significant differential expression of miRNAs known to have a role in brain development and function, clustered within the *Dlk1-Dio3* imprinted domain [300]. Furthermore, more recent profiling of miRNAs in poly(I:C)-exposed mice offspring has shown significant changes in the hippocampus, including miR-877, miR-466 and miR-16 [301,302]. Moreover, recent evidence suggests that tRFs, a type of small ncRNAs that are cleaved from tRNAs, are also disrupted in response to mIA [287,303]. Notably, Su et al. [303] showed that both microRNAs and tRFs, including tRNA halves, were transiently altered in the maternal-fetal interface (placenta/decidua), with the most significant changes found 3 h post poly(I:C) injection. Taken together, these data suggest that changes to short ncRNAs may represent an understudied underlying mechanism responsible for mIA-altered gene expression in the developing brain with emerging data suggesting that effects on placental ncRNAs may also contribute to the fetal response to mIA.

## The COVID-19 pandemic and early childhood neurodevelopment: impact of maternal infection

### COVID-19 and pregnancy

Pregnant women are a high risk group for severe acute respiratory syndrome coronavirus 2 (SARS-CoV-2) infection as the physiological changes in the immune system during pregnancy could affect the progression of the disease [304]. The clinical manifestations noted in those testing positive for SARS-CoV-2 in hospital indicated that most women during pregnancy had a mild or moderate illness or were asymptomatic, though a small proportion had a more severe illness in the late second or third trimester of pregnancy [305,306]. Adverse pregnancy outcomes such as preeclampsia, small-for-gestational age and fetal distress were diagnosed in more women with SARS-CoV-2 infection, than in those without, and preterm delivery appeared to reflect the severity of their illness and carried a potential risk for neonatal morbidity and admission to a specialist neonatal intensive care unit [307,308]. As already outlined, some viral infections acquired during pregnancy can have damaging effects on the developing fetal brain, also postulated for SARS-CoV-2 infection [309,310]. Emerging evidence of an increase in neurodevelopmental impairments in the first few months of age among infants born during the coronavirus disease 2019 (COVID-19) pandemic, caused by the SARS-CoV-2 virus, has drawn attention to the prenatal exposure to this maternal infection [309]. In addition, the stress induced by exposure to the rapidly changing pandemic environment, including social distancing, financial strain, loss of childcare, school closures and food insecurity, has impacted on normal family life as well as mother-infant bonding, especially in under-resourced minorities [311]. It has been estimated that nearly 20 million babies per year could have been exposed globally to maternal COVID-19 infection *in utero* when infection was prevalent, with acknowledgement of the potential for neurodevelopmental morbidity in the offspring [309,310]. Indeed, a UK cohort study showed that in children hospitalised with neurological and psychiatric manifestations and testing positive for SARS-CoV-2, the central nervous system was particularly vulnerable with a high incidence of neuroimmune disorders reported related to this infection [312].

Vertical transmission of the virus from mother to fetus is uncommon but has been reported in a few cases [310,313], although its clinical significance in small numbers of infants previously based on case reports is not clear [305,314,315]. In a systematic review of the available literature, the vertical viral transmission rate was estimated as 0.63% and no structural congenital anomalies have emerged in newborns of SARS-CoV-2 positive mothers [316]. Hence, there is little evidence that SARS-CoV-2, can cross the human placenta [304,313,317], but the placentas of infected mothers do, however, display indicators of inflammation such as infiltration of maternal immune cells [313]. Moreover, the mIA response to SARS-CoV-2 infection, even in cases of mild disease, has been proposed to play a key role in affecting fetal development, including programming of the developing brain, through effects on the placental and immature blood-brain barriers rather than a direct effect of viral infection [318–321].

### Newborn infants: prenatal exposure to maternal SARS-CoV-2 infection

SARS-CoV-2 uses the angiotensin-converting enzyme 2 (ACE2) receptor as the primary receptor and neuropilin-1 (NRP1) as a co-receptor together with two proteases, transmembrane protease serine 2 (TMPRSS2) and cathepsin L (CTSL), and the pro-protein convertase FURIN, for cell entry to gain access to the host cell [313,322], leading to acute infection. It is intriguing that these entry receptors are highly expressed in the first and second trimester placentas, whereas at term, placental expression is lower [313], perhaps providing an access route for placental viral uptake, where placental viral load appears to correlate with clinical severity of maternal disease [313]. Interestingly, viral RNA and protein detected in human placenta was restricted to the epithelial layer of the syncytiotrophoblast that is in direct contact with maternal blood, and the underlying cytotrophoblast layer, with no evidence of progression beyond this into the villous core containing fetal capillaries [313].

The maternal immunological profile associated with SARS-CoV-2 infection in pregnancy and a potential impact of mIA on neurodevelopment in infants born during the pandemic has gained considerable interest [309,310,320]. SARS-CoV-2 infection in pregnancy was associated with an inflammatory response in the maternal circulation and at the maternal–fetal interface [321,323]. The possibility that the maternal immune response to an acute SARS-CoV-2 infection could cause damage to the fetal brain through inflammatory processes is supported by animal studies investigating links between mIA-driven risk for NDD conditions such as SZ and ASD [8,318,324,325], and as outlined above. This may impact genes that promote early brain development and function through dysregulated epigenetic mechanisms [44,142,310], as discussed in the previous section.

Maternal inflammatory cytokines and chemokines, including IL-6, CXCL10 and IL-8, were found to be increased among both early (first/second trimesters) and late (third trimester) pregnancy SARS-CoV-2 infections and were also present in cord blood [319]. A more extensive immune profiling, in largely asymptomatic women, also found IL-10, IL-15 were significantly elevated in maternal blood [321]. Infants born to mothers testing positive for SARS-CoV-2 even had raised IL-8 concentrations in cord blood suggesting that this maternally generated cytokine is capable of crossing the placenta and entering the fetal circulation, while viral IgM antibody was undetectable in cord blood indicating that acute fetal infection did not occur [321]. The placentas of infants born to infected mothers had altered immune cell type ratios and altered immune activity as measured by immunophenotyping in the placenta and cord blood [321], and as already mentioned, there is a profound migration of maternal monocyte/macrophages and T cells into the maternal blood spaces [313]. Interestingly, SARS-CoV-2 infection was associated with the dysregulation of transcripts in maternal blood; those involved in humoral responses such as complement activation, adaptive immune responses, and immunoglobulin-mediated immune response were upregulated, whereas those enriched for phagocytosis and extracellular matrix organization were downregulated [321]. Similarly, SARSCoV-2 infection caused transcriptional dysregulation in cord blood cells including immune cell response [321]. Of note, a strong placental inflammatory response was seen in infected samples with profound increases in the expression of proinflammatory cytokines, chemokines, and other inflammation-related genes when human placental cell clusters were treated *ex vivo* with SARS-CoV-2, including upregulated IL-6, TNFα, IL-8 and 1L-19, with IL-6 and IL-10 signalling pathways appearing in the top canonical biological pathways affected [313]. The cytokine IL-6, in particular may have a potential impact on fetal health as it is a key intermediary in pathways whereby mIA alters fetal brain development in animal studies [86]. In pregnant women with SARS-CoV-2 infection, raised IL-6 concentrations have been correlated with the severity of infection [326] and IL-6 is one of several cytokines linked with preterm labour associated with maternal infection [327], and as already discussed, is probably capable of crossing the placenta [99,110]. Furthermore, IL-6 has also been associated with impaired neurodevelopment in children with cerebral palsy [328,329].

Collectively, these findings suggest that the placental transcriptome is affected by maternal SARS-CoV-2 infection with induction of inflammatory markers, also apparent in the fetal/neonatal immune system even if an acute fetal infection is unlikely to have occurred. Furthermore, the perturbations in immune cell populations and cytokines present in cord blood, in the absence of fetal infection, appear to enhance the potential for these cells to produce cytokines, not only in neonates born to mothers with recent or ongoing infection, but also in those from recovered mothers, suggesting *in utero* priming of the immune response is imprinted on the neonate [330]. Although the placenta appears to serve a protective role in preventing vertical transmission of SARS-CoV-2, the possibility exists that infection of the placenta, and downstream induced placental and fetal inflammatory responses, including placental-related vascular injuries, fetal growth restriction and fetal brain lesions, as detected by prenatal MRI scans [331], could have lasting developmental effects on infants, highlighting the need for the future assessment of children for developmental defects.

### Prenatal exposure to maternal SARS-CoV-2 infection and early childhood development

In infants born during the pandemic, emerging evidence of neurodevelopmental delay has raised concerns about whether prenatal exposure to maternal SARS-CoV-2 infection, the immune response to it, or the mother's psychological condition contribute to this delay [332,333]. However, the stresses related to exposure to the pandemic on family life including disruption of schooling and their impact on early childhood development are also recognised [311,334]. In the USA, a retrospective cohort study investigated the effects of prenatal exposure to maternal SARS-CoV-2 infection on infant development [335]. Preterm births occurred in a higher proportion of mothers with the infection. By age of 1 year, 6.3% of 222 infants exposed prenatally to maternal SARS-CoV-2 had a neurodevelopmental impairment compared with 3.5% of 7550 unexposed infants, with an increased frequency of specific developmental disorder of motor function, expressive language disorder, unspecified developmental disorder of speech and language, or other disorders of psychological development in the infection-exposed group. After statistical adjustment for race, ethnicity, insurance status, offspring sex, maternal age and preterm birth, a higher rate of neurodevelopmental impairment persisted among infants of mothers who had infection during pregnancy. The observed neurodevelopmental impairment rate associated with prenatal exposure to SARS-CoV-2 infection appeared to be enhanced by preterm birth, though this could not be entirely explained by prematurity.

A recent study in the USA [336] compared infant development in those exposed and unexposed prenatally to maternal SARS-CoV-2 infection, and in infants born at the same hospital before the pandemic (a historical cohort) that attended a Well Baby Clinic. Infant development was assessed using the Ages & Stages Questionnaire 3^rd^ edition (ASQ-3) in 114 infants exposed to maternal SARS-Co-2 infection and in 141 unexposed infants born during the pandemic. This study found no significant differences in any of the five subdomain scores of ASQ-3, including communication, gross motor, fine motor, problem solving, or personal social skills at age 6 months. Statistical adjustment for gestational age at birth, infant sex, age at assessment, and maternal race, ethnicity, age at delivery, educational level, parity, mode of delivery and neonatal intensive care resulted in no significant differences between the study groups. After excluding infants born preterm or admitted to a neonatal unit, the ASQ-3 scores in a subset of 227 infants born during the pandemic, regardless of maternal SARS-CoV-2 status, were significantly lower in gross motor, fine motor and personal-social domains when compared with those in 62 infants in the historical cohort. These findings raised the possibility that pandemic-related stresses were implicated in an underlying mechanism that impacted early childhood development. The potential effects of job losses, food insecurity and loss of housing were suggested as they could have affected normal family life and infants growing up in this environment had shown differences in developmental outcome [336].

The results of the two studies indicate a wealth of factors can underscore how the COVID-19 pandemic may affect child development. Whether these outcomes reflect causal associations of prenatal exposure of the fetal brain to maternal infection and the impact of immune activation or mechanisms, or consequences related to societal changes and stresses to family life having an impact on early development [337], remains to be fully defined. There may have been differences in the timing of maternal infection during pregnancy and in the severity of illness. Notwithstanding these issues, others have found an increased risk of developmental delay in infants of mothers who had SARS-CoV-2 infection in the first or second trimester of pregnancy than in those whose mothers had infection in the third trimester [338], perhaps related to the expression of proteins that permit SARS-CoV-2 placental entry [313], as discussed above. Also, infants exposed prenatally to maternal SARS-CoV-2 had a lower motor optimality score [339] and infants screened during the pandemic scored lower in communication as compared to those screened before the pandemic, and those exposed prenatally to maternal SARS-CoV-2 scored lower in fine motor skills [340].

In demonstrating that exposure to the pandemic or prenatal exposure to maternal SARS-CoV-2 may contribute to the cause of NDD in early childhood, these associations do not indicate whether the NDDs are transient or lasting changes in central nervous system function. The gestational timing of maternal SARS-CoV-2 infection, the severity of infection, or the identity of the different SARS-Cov-2 variants could potentially contribute to developmental outcome [341]. But collectively, they support the need for long term follow-up, generating hypotheses and raising questions for future studies related to causality. A better understanding of the underlying causes would help to determine how best to intervene to mitigate the effects of the pandemic, and in doing so, achieve long term benefits to support the health and wellbeing not only of children, but also their progression to adulthood when other environmental triggers may reveal underlying neuropathology.

## Concluding remarks

**Figure 1:**
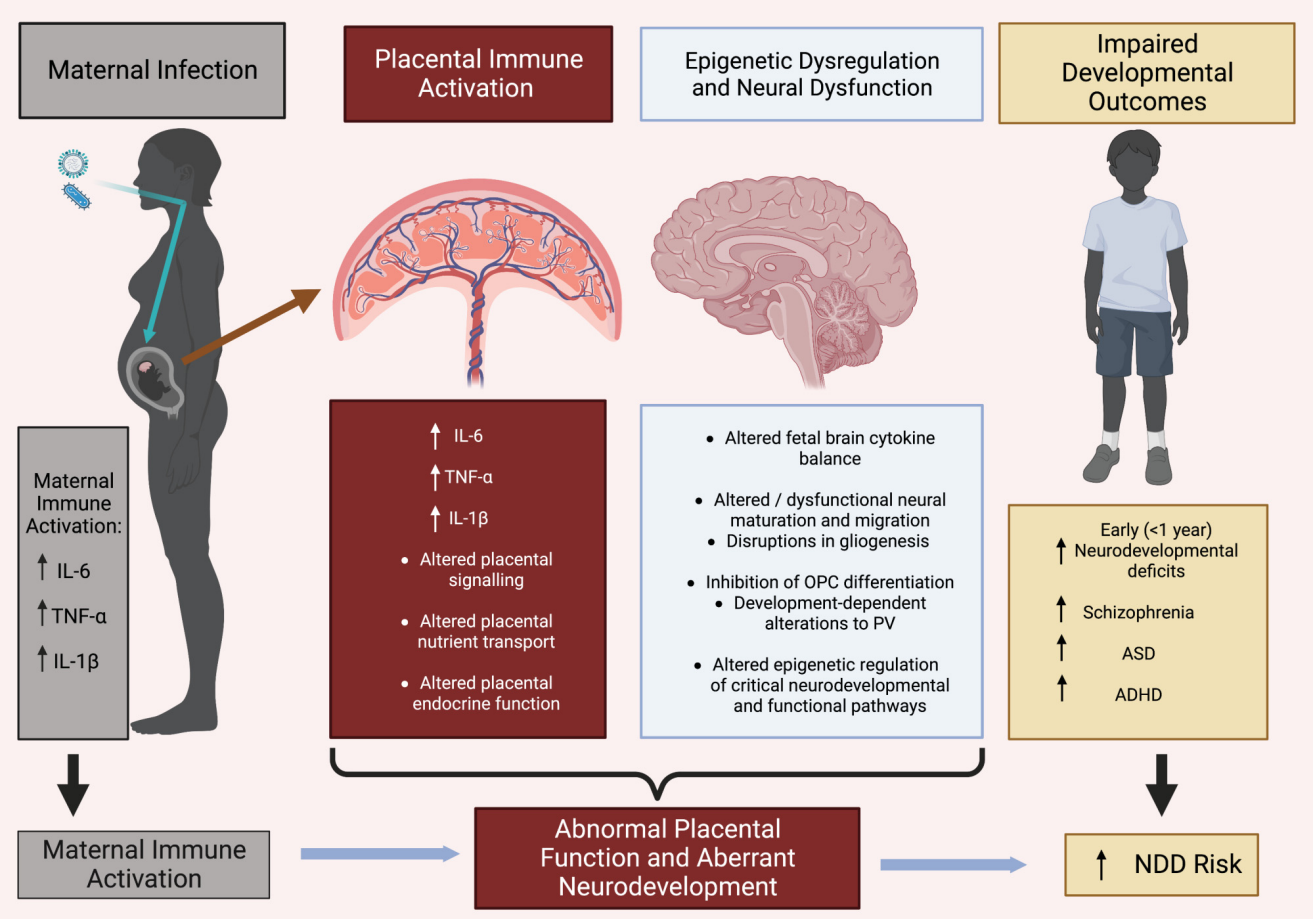
Infection in pregnancy and implications for offspring neurodevelopment Maternal infection during pregnancy evokes maternal immune activation and increased systemic proinflammatory cytokine release. This results in the activation of maternal monocytes at the placental interface, resulting in placental immune activation and inflammation, associated with an increased placental concentration of proinflammatory cytokines. This leads to altered placental signalling and function and subsequent fetal brain neuroinflammation and disrupted cytokine balance, which affects epigenetic regulation of key neurodevelopmental pathways and impacts on neurodevelopmental signalling, causing microglial priming, disturbed neural progenitor cell proliferation, impaired neuronal migration and synaptogenesis, along with reduced myelin plasticity, which, together, can result in altered offspring neurodevelopment. Offspring affected by maternal immune activation have an increased risk of developing neurodevelopmental disorders (NDDs), including schizophrenia, autism spectrum disorder (ASD) and attention-deficit hyperactivity disorder (AHDH). *Created with BioRender*.

Pregnant women comprise a group that have an enhanced risk of developing severe illness in response to infection. Stimulated maternal immune responses to infection, with accompanying mIA and the generation of proinflammatory cytokines and chemokines, raises the possibility for detrimental impacts also on fetal development (Figure 1). In particular, neural development presents a protracted developmental window susceptible to environmental stressors, as it involves an intricate and highly complex orchestration of staged events, both spatially and temporally, over both the prenatal and postnatal phases of development. During normal brain development, cytokines act as critical signalling molecules, so a perturbed local cytokine environment and/or the existence of neuroinflammation, is likely to have an influence on neurodevelopmental events and underlying neurodevelopmental trajectory, with consequences for NDD risk and predisposition to aberrant neuropsychiatric characteristics.

In order to devise therapeutic strategies to mitigate against this, it is imperative to delineate fully the neurodevelopmental mechanisms that are impacted by mIA, and define susceptible molecular loci that are affected, to understand comprehensively how mIA-evoked responses lead to enduring neuropathology in the offspring. In this context, mIA models, mostly in mice and rats, have been developed to simulate infection during pregnancy using mimetics such as LPS and poly(I:C). These have been invaluable in determining the scope of mIA responses in the mother, whilst providing a platform for investigating concomitant or subsequent changes within the fetal compartment, both at the placental interface and within the developing fetus. They have also afforded the benefit of being able to examine the pathogenesis of NDDs along a longitudinal developmental timeline into adulthood.

These studies have yielded a bounty of mechanistic insights, but the collective evidence supports a scenario whereby the mIA-stimulated elevation in maternal systemic cytokine concentrations is accompanied by joint inflammatory responses in the placenta and fetal brain (Figure 1). Activation of maternal immune cells within the placenta leads to an upregulated synthesis of cytokines and chemokines, resulting in raised placental concentrations. This response acts as an autocrine/paracrine signal to initiate multiple signalling cascades that alter the placental transcriptome and function of fetal trophoblast cells within the placenta. This can plausibly affect multiple downstream functional axes, including placental development, the capacity to provide nutrients or precursors that are crucial for normal fetal development, and/or its regulation, which collectively are likely to cause dysfunction of developmental processes and derangement of normal fetal physiological homeostasis (Figure 1). Within this setting, the activation of a variety of fetal signalling cascades, including immune response pathways, is likely to be invoked, consistent with the extensive changes found to the neural transcriptome and epigenome in the brains of mIA-exposed fetuses, at diverse molecular loci [44,163]. Obviously, the gestational timing of such perturbations and their duration will dictate the scope of neural developmental and functional impact, along with the plasticity of fetal adaptive responses to attenuate dysregulated function. Prenatal cytokine-driven perturbation in the developing brain alters neurogenesis, neuronal proliferation, migration, signalling and survival as well as glial and astrocyte activation (Figure 1). Such events are likely to be modulated by ‘placenta–fetal brain’ regulatory signalling cross-talk to prime such dysfunction. Hence, the concept that the ‘placenta is a window to the brain’ [140] is bolstered, and a more comprehensive elucidation of the mechanistic changes that mIA evokes to placental molecular function, and how these ‘program’ an aberrant neurodevelopmental trajectory that predisposes to an increased risk of NDDs in later life (Figure 1), will not only assist in therapeutic discovery, but may also be revelatory within the post-COVID-19 pandemic era.

Hence, the attainment of mechanistic insights and a better understanding of the mIA-related responses to SARS-CoV-2 affecting maternal health during pregnancy and the longer term impact for the mother and her child's development, allows the overlay of such knowledge to social medicine and public health, with the influences of stress, ethnicity, socio-economic position, and changing family life in the pandemic environment to be more completely understood. Further, how these collective influences impact on child development and relate to the disconcerting increase observed in NDDs highlights lessons to be learnt in meeting inevitable future pandemics.

## Data Availability

Data sharing is not applicable as this is a review article.

## Open Access

Open access for this article was enabled by the participation of University of Manchester in an all-inclusive *Read & Publish* agreement with Portland Press and the Biochemical Society under a transformative agreement with JISC.

## Abbreviations

ACE: angiotensin-converting enzyme
ADHD: attention-deficit hyperactivity disorder
ASD: autism spectrum disorder
ASQ-3: Ages & Stages Questionnaire 3^rd^ edition
BDNF: brain-derived neurotrophic factor
COVID-19: coronavirus disease 2019
E/I: excitatory/inhibitory
GABA: γ-aminobutyric acid
GAD: glutamic acid decarboxylase
GD: gestational day
H3: histone 3
H4: histone 4
JZ: junctional zone
LPS: lipopolysaccharide
LZ: labyrinth zone
MAPK: mitogen-activated protein kinase
mIA: maternal immune activation
miRNA: microRNA
ncRNAs: non-coding RNAs
NDD: neurodevelopmental disorder
NFκβ: nuclear factor kappa light chain enhancer of activated B cells
NPC: neural progenitor cell
OPC: oligodendrocyte progenitor cell
PD: postnatal day
poly(I:C): polyinosinic:polycytidylic acid
PPI: prepulse inhibition
PVI: parvalbumin interneurons
SARS-CoV-2: severe acute respiratory syndrome coronavirus 2
STAT3: signal transducer and activator of transcription 3
SVZ: subventricular zone
SZ: schizophrenia
TLR: toll-like receptor
TPH: tryptophan hydroxylase
tRFs: transfer RNA (tRNA)-derived fragments
VZ: ventricular zone
